# Breast Microcalcification Diagnosis Using Deep Convolutional Neural Network from Digital Mammograms

**DOI:** 10.1155/2019/2717454

**Published:** 2019-03-03

**Authors:** Hongmin Cai, Qinjian Huang, Wentao Rong, Yan Song, Jiao Li, Jinhua Wang, Jiazhou Chen, Li Li

**Affiliations:** ^1^School of Computer Science and Engineering, South China University of Technology, Guangzhou 510000, China; ^2^Guangdong Provincial Key Lab of Computational Intelligence and Cyberspace Information, South China University of Technology, Guangzhou, China; ^3^Sun Yat-sen University Cancer Center, State Key Laboratory of Oncology in South China, Collaborative Innovation Center for Cancer Medicine, Guangzhou, Guangdong 510060, China; ^4^Medical Imaging Center, Shenzhen Hospital of Southern Medical University, Shenzhen, Guangdong 518101, China

## Abstract

Mammography is successfully used as an effective screening tool for cancer diagnosis. A calcification cluster on mammography is a primary sign of cancer. Early researches have proved the diagnostic value of the calcification, yet their performance is highly dependent on handcrafted image descriptors. Characterizing the calcification mammography in an automatic and robust way remains a challenge. In this paper, the calcification was characterized by descriptors obtained from deep learning and handcrafted descriptors. We compared the performances of different image feature sets on digital mammograms. The feature sets included the deep features alone, the handcrafted features, their combination, and the filtered deep features. Experimental results have demonstrated that the deep features outperform handcrafted features, but the handcrafted features can provide complementary information for deep features. We achieved a classification precision of 89.32% and sensitivity of 86.89% using the filtered deep features, which is the best performance among all the feature sets.

## 1. Introduction

Breast cancer is the most common cancer affecting women's health. Early detection of breast cancer has been shown to increase the survival rate, thereby significantly increasing patients' lifespan [1]. Mammography is a very popular noninvasive imaging tool with low cost compared with other advanced equipment, such as computed tomography. It is widely used to diagnose breast disease at an early stage due to its high sensitivity. Therefore, it is frequently used as a tool for early screening.

During mammography screening, the presence of breast microcalcifications (MCs) is a primary risk factor for breast cancer. Breast calcifications in the early stages of breast cancer appear like scattered spots in the mammographic image that range from 0.1 to 1.0 mm in size [2]. Previous studies have found that MCs associated with malignant lesions tend to be smaller in size, greater in amount, and are more densely distributed since they occur within the milk ducts and other associated structures in the breast and follow the ductal anatomy [3]. Because a high correlation has been observed between the appearance of calcification clusters and pathology results, the MCs provide a standard and effective way for the automated detection of breast tumors.

Besides, large-scale genome-wide association studies (GWAS) have proved to be a strong support for identifying disease risk pathways [4]. Experimental results provide clinically useful clues about the link between these risk genes and MS susceptibility in the Chinese population. The study of [5] demonstrates convincingly that the genetic predisposition for development of AD is rooted in the immune system, rather than in neuronal cells in some degree.

Traditionally, radiomics diagnosis systems consider the mammographic diagnosis to be a pattern recognition and classification problem. Therefore, classical image processing and machine learning techniques were combined to discriminate the suspicious MCs and differentiate their types. Generally, a standard diagnosis system consists of image segmentation and feature extraction for calcification and classification [6]. The feature extraction aims to characterize calcification with quantitative descriptors. The popular features include low-order statistics, such as shape [2, 7], and high-order statistics, such as morphological [8] and texture features [9]. However, because of the large homogeneous condition of breast cancer and various image settings, few universally reliable and robust imaging features have been reported to perform equally well on different mammography datasets. In addition, there are other pitfalls within traditional approaches. For example, there is no causal relationship between the classification model and manually extracted features. In some images, morphological features cannot be acquired when the calcification of interest only occupies a few pixels.

Recently, deep learning models with the convolutional neural network (CNN) have gained wide attention because of their efficiency in obtaining automatic informative feature representation and high accuracy by unifying the classification and feature representations as a whole. It has been successfully evaluated in medical image analyses [10]. For example, previous studies have used a hybrid CNN to achieve high mass and pathologic classification [11, 12]. In addition, a convolutional sparse autoencoder has been previously employed to form a CNN, which obtained good results in breast density segmentation for mammography risk scoring [13].

Inspired by the promising applications of deep learning models in medical image diagnosis, one popular CNN model was tailored to provide an automatic and comprehensive characterization of MCs, resulting in a deep feature representation. For comparison, traditional manual image descriptors were used to extract handcrafted features. To further exploit the merits of both types of feature characterization, we use two methods to achieve it. One is the combination of deep features and handcrafted features, and the other is a novel feature selection strategy using the proposed CNN structure to achieve full usage of the traditional descriptor.

The major contributions of our work are as follows:

1. We proposed a fully automatic pipeline to detect, analysis, and classify microcalcification on an empirical mammography dataset. The tested dataset contains 990 images. All images are confirmed with biopsy to have the lesion types.
2. We applied both handcrafted and deep learning-extracted image features to compare their performances. To exploit the merits of the two methods, the two types of features were also fused together to enhance the classification performances.
3. We achieved high accuracy of classification on the dataset.

## 2. Literature Review

The prognostic decision on the type of microcalcification clusters was mainly focusing on extracting informative handcrafted features and then building a highly discriminative classier on it. In [14], the author classified clustered microcalcifications (MCCs) as benign or malignant using a set of wavelet features, and the classifiers were used, namely, Artificial Neural Network (ANN) and SVM. In [15], the authors performed the microcalcification characterization using morphologic features which can be used to feed a neuro-fuzzy system to classify the detected breast microcalcifications into benign and malignant classes. Kooi et al. [12] used textural features and interest points or corners to train a random forest classifier to achieve microcalcifications diagnosis.

Recently, deep learning model CNN has gained much popularity due to its high accuracy, great power, and flexibility. Deep features have been applied to the classification of microcalcification clusters. Becker et al. [16] showed that current state-of-the-art networks for general image analysis could detect cancer in mammographies with similar accuracy to radiologists. This motivated us to explore deep learning as the basic framework for the classification of microcalcification. In [17], the authors presented an automated CAD system with minimal user intervention that can detect, segment, and classify breast masses from mammograms. In addition, there are many methods which rely on combining different information. For example, the study in [18] introduced a novel system that integrates several modules including a breast segmentation module and a fibroglandular tissue segmentation module into a modified cascaded region-based convolutional network. The study of Jiao et al. [19] is closely related to our work. The authors designed a deep feature-based framework combining intensity information and deep features automatically extracted by the trained CNN. In [20], deep learning was used for the discrimination of breast cancer with microcalcifications. Inspired by Faster-RCNN, Ren et al. and Ribli et al. [21, 22] proposed a CAD system which achieved the state-of-the-art classification performance on the public INbreast [23] database. Similarly, we fine tuned the model through transfer learning to overcome the problem of overfitting. In [24], the authors developed a context-sensitive deep neural network (DNN) for microcalcification detection. In this paper, we explored the ideas above, i.e., the deep learning model and the combination of different information.

## 3. Materials and Methodology

### 3.1. Dataset

**Figure 1:**
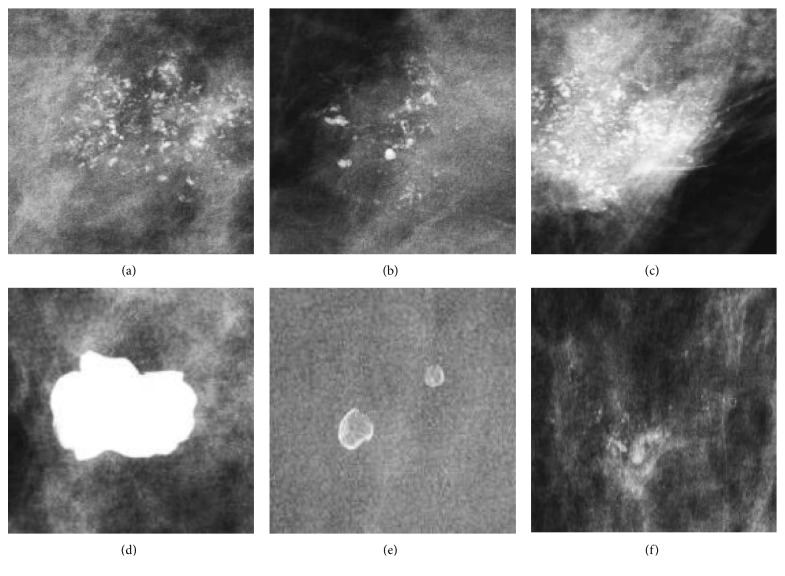
Examples of malignant and benign lesions. (a)–(c) Images showing malignant lesions, including ductal carcinoma in situ, invasive ductal carcinoma, and mixed type. (d)–(f) Images showing benign lesions, including a benign lesion after follow-up, inflammation, and fibrocystic mastopathy.

The datasets were collected at two medical institutions, the Sun Yat-Sen University Cancer Center (SYSUCC) and Nanhai Affiliated Hospital of Southern Medical University (Foshan, China). There are 749 samples collected from SYSUCC, and the remaining 241 samples are from Nanhai Affiliated Hospital of Southern Medical University. During the learning process, the two datasets were mixed to enhance the robustness of the prognostic of the model. The datasets consist of 990 images (types: full-field digital mammography, resolution: 1912 × 2294, bit-depth: 8) from 328 breast lesion cases (age 21–73 years, mean 45 years). Within the lesion images, 540 images presented malignant masses and 450 were benign lesions, as proved histopathologically by biopsy.  Table 1 shows the number of mammograms for all eight types of malignant pathologies and six different benign pathologies. In Figure 1, we illustrate some examples of malignant and benign lesions. Considering the limited data, we add simple rotation (0°, 45°, 90°, and  135°) to the ROIs attained by segmentation as data augmentation. Thus, of the 990 images, 3564 ROIs from 891 images were used as the training set and 99 ROIs from 99 images (45 benign and 54 malignant) as the test set, as shown in Table 2.

### 3.2. Methods

**Figure 2:**
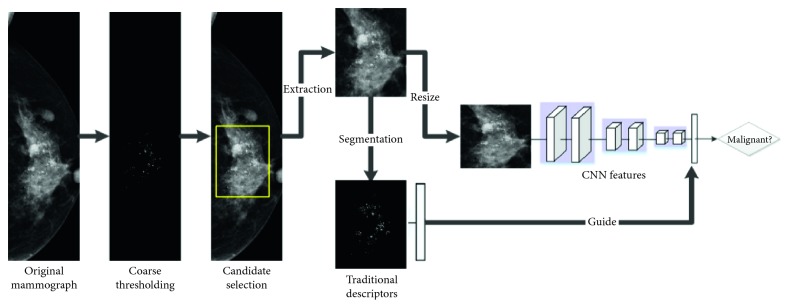
Workflow diagram for the classification of MCs. The calcification areas of interest were first detected and then characterized by both deep learning and traditional manual descriptors. The results from the two feature types were evaluated and compared independently. To enhance the diagnostic performances, the two feature types were further combined or filtered to accomplish a complete characterization of the MCs.

Our method consists of three major steps. In the first step, the suspicious region of interested calcification (ROIC) area was extracted by an automatic image preprocessing method. In the second step, radiomics feature was learned both by handcrafted [25] and fine-tunied pretrained CNN model. The handcrafted features include the first-order statistical, morphological, and texture features. Finally, various classifiers were trained and evaluated by using benchmark support vector machine (SVM) model based on the deep features, handcrafted features, combined features, and filtered features individually. A schematic diagram of the proposed method is illustrated in Figure 2.

#### 3.2.1. ROIC Extraction

**Figure 3:**
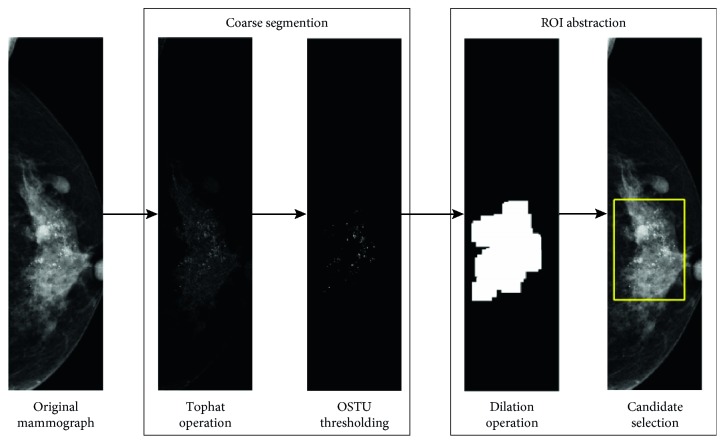
Workflow diagram for ROI extraction. A coarse segmentation scheme was performed using traditional morphological filters. Then the locations showing the most MCs were used for ROI extraction.

**Figure 4:**
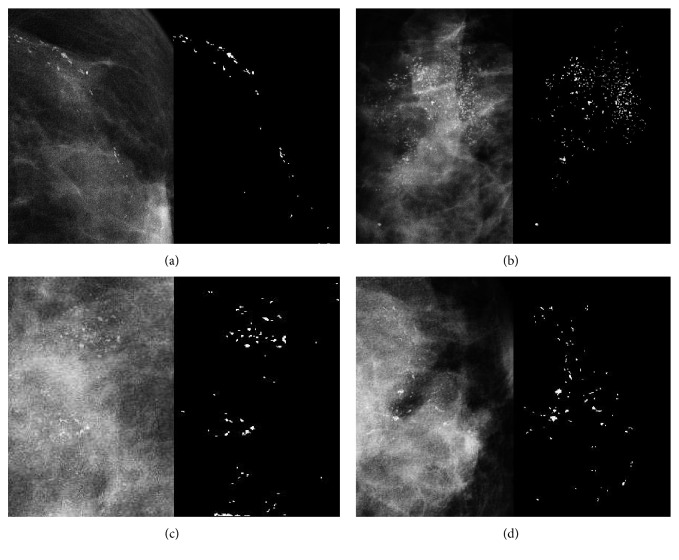
Experimental results on calcification extraction.

The ROI extraction aims to standardize the mammographic images and extract calcification areas as regions of interest. To make full use of all information of regions with MCs, we extracted the ROI using a coarse segmentation scheme. We firstly applied morphological erosion to a structure element radius of 100 pixels to remove the pixels close to the breast outline. Then a morphological top-hat filtering with a ball structural element with a radius of 8 and a height of 100 pixels was applied. The resulting grayscale image was converted into a binary image by Otsu thresholding [26]. The binary image was finally dilated with a disk-shaped structural element with a radius of 100 pixels, and the maximum connect region was considered as the calcification area. An illustrative example is shown in Figure 3. The most calcification areas can be accurately segmented, as shown in Figure 4.

#### 3.2.2. Deep Feature Extraction

We built a deep CNN framework to extract the deep features. The proposed framework was similar to an early successfully tested network, which was designed for nature image recognition [27]. The proposed CNN architecture employed the same 5 convolutional layers AlexNet as the base structure for tuning feature representation. It comprises five convolutional layers of {96, 256, 384, 384, 256} with a kernel size of {11, 5, 3, 3, 4}, respectively. Each convolutional layer in the network consists of a number of convolution filters and rectified linear unit activation. Three max-pooling layers are, respectively, constructed to follow the two previous and the last convolutional layers to reduce data dimension. With the help of multiple layer architecture and drop-out strategy to alleviate the overfitting, the network can obtain robust and spatial invariant features. Note that we did not adopt a deeper architecture because our preliminary experiments using 16 layers [28] were less satisfactory than the AlexNet-like architecture [27] used in the present study. The deeper architecture was pruned to result in overfitting.

In a further cautious step to overcome the problem of overfitting, we borrowed the “off-the-shelf” model from ImageNet [29] and fine-tuned it through transfer learning for our purpose. Although there are huge disparities between medical images and natural images, CNN was trained on the large-scale ImageNet [29], which has the capacity to describe the outline and other details, was transferred to make our task more effective.

To extract the feature representation by CNN, the tested images were propagated through the CNN. The resultant penultimate layer activations of the whole network were used as feature representations by the CNN.

#### 3.2.3. Handcrafted Feature Extraction

The majority of the traditional prognostic systems rely on accurate manual calculations to determine microcalcification (MC) features. Popular radiomics features including statistical measurements in the ROI, such as spatial and textural features [20, 30–32], morphological features, and textural features have been reported to have the best performance for mass classification. In our study, 200 morphological features were computed from the binary lesion shape, and 352 texture features were extracted from the segmented ROI image. The names of each quantitative descriptors and their respective computational technique are summarized in the supplementary available (here).

#### 3.2.4. Canonical Correlation Analysis to Fuse Both Handcrafted and Deep Features

We used canonical correlation analysis (CCA) [33] to fuse both the obtained CNN features and the handcrafted features. The primary purpose of CCA is to exploit the merits of both feature types, thus enhancing the diagnostic performances.

Mathematically, *X* ∈ *R*_*p*×*n*_ is the representation of deep feature and *Y* ∈ *R*_*q*×*n*_ can be one or all of the handcraft descriptors. The CCA aimed at finding linear weighting vectors *w*_*X*_, *w*_*Y*_ to maximize the pair-wise correlations across the two data sets:

(1) $$\begin{array}{c} \begin{array}{cc} \underset{w_{X},w_{Y}}{\max} & \text{corr}\left(X^{*},Y^{*}\right)=\frac{\mathrm{cov}\left(X^{*},Y^{*}\right)}{\mathrm{var}\left(X^{*}\right)*\mathrm{var}\left(Y^{*}\right)},\\ \text{subject to} & \mathrm{var}\left(X^{*}\right)=1,\\ & \mathrm{var}\left(Y^{*}\right)=1, \end{array} \end{array}$$

where *X* ^*∗*^=*w*_*x*_^*T*^ *X* and *Y* ^*∗*^=*w*_*y*_^*T*^ *Y*.

Once the weighting vector of first canonical variate pair was obtained, we retained the deep features that top 10% high coefficients in weighting vector correspond to. The filtered deep features have high correlations with handcrafted features.

### 3.3. Prognostic Classifiers Building

Throughout our study, the support vector machine (SVM) model was borrowed as a base classifier to evaluate the diagnostic performance of the features. The SVM model has been widely used as a benchmark model for image classification [34, 35]. The basic idea of the SVM model is to maximize a linear hyperplane spanned by samples in support positions. The hyperparameters used in the SVM were fine-tuned to obtain the best performance. For the SVM classifier, RBF (radial basis function) kernel function was used.

Several popular quantitative measurements were used to quantify the classification performances. The measurements included accuracy, precision, sensitivity, and specificity, defined as

(2) $$\begin{array}{c} \text{accuracy}=\frac{\text{TP}+\text{TN}}{\text{TP}+\text{TN}+\text{FP}+\text{FN}},\\ \text{precision}=\frac{\text{TP}}{\text{TP}+\text{FP}},\\ \text{sensitivity}=\frac{\text{TP}}{\text{TP}+\text{FN}},\\ \text{specificity}=\frac{\text{TN}}{\text{TN}+\text{FP}}, \end{array}$$

where true positive (TP) was the number of malignant samples that were correctly classified and true negative (TN) was the number of nonmalignant samples that were correctly classified into benign. The false-positive (FP) and false-negative (FN) values were defined similarly. Except for those measurements mentioned above, we also adopt the area under the ROC curve (AUC) as the measurement.

## 4. Experiment Methodology

The Caffe framework [36] was employed to build and fine-tune the network in our experiment. Since the trained model in ImageNet by AlexNet was designed for nature images with three channels, the mammography samples in our study were converted into three channels by copying grayscale to each channel. All CNN layers except the last and the penultimate layer were inherited from the trained model in AlexNet for fine-tuning. The learning rate in the training stage was initialized at 0.01 and the training stage continued for 300 epochs. Once the transfer learning process was completed, the neurons in the penultimate layer of the whole network were extracted as representative features for the tested mammographic images. The model was trained and tested using 10-fold cross validation on the dataset. The parameters (i.e., learning rate, batch size, and epochs) were fine-tuned in each round of the cross validation.

## 5. Experimental Results

The classification performances of MCs are summarized in Table 3. The table provides the average and the standard deviation for different measurement indexes. Compared with the manual features, the CNN features achieved superior performance in terms of both accuracy and sensitivity. The diagnostic accuracy for CNN was 0.8768 compared with 0.8667 for manual features. In addition, the combined features and filtered features outperform CNN features. Most significantly, the filtered feature can improve the classification more than CNN feature, which implies that the CNN feature is not the most perfect for classification and it can perform better by combining the conventional feature or being filtered by conventional feature. Compared with the simple combination, the performance of the proposed CCA scheme obtained better diagnostic power from AUC (0.9398 ± 0.0242 versus 0.9379 ± 0.0237). Morphological features contribute the most among all the conventional features. The CNN feature filtered by morphological features obtained the highest accuracy (0.8859 ± 0.0363). We also performed the whole experiment based on different distributions of data to explore the impact of them. The obtained experimental results demonstrate that the superior performance of distribution for training and testing data is 90% and 10% compared with other distributions, as summarized in Table 4.

For an easy comparison of the proposed method, we have cited the experimental results obtained by four different techniques on another mammography dataset, BreaKHis, as shown in Table 5. The reported accuracies ranged from 83.3% to 99.4%. We did not apply our method on the dataset since it contains few mammographies with calcifications.

**Figure 5:**
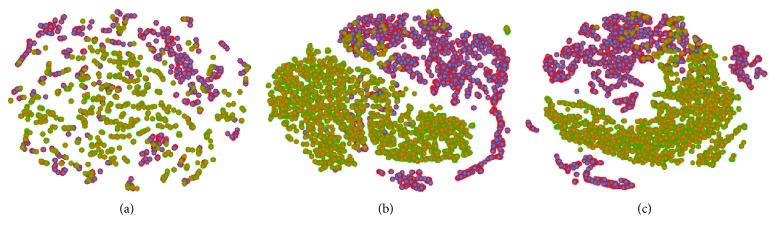
(a–c) *t*-SNE maps of handcrafted features, deep features filtered by handcrafted features, and deep features. The benign and malignant samples are highlighted in red and green, respectively. The filtered features and deep features are largely categorized into two clusters.

We employed *t*-distributed stochastic neighbor embedding (*t*-SNE) [41] to visualize different feature sets by locating each sample in a two-dimensional map in Figure 5. The manual features are distributed unevenly, thus making it challenging to separate as shown in Figure 5(a). The distribution of the filtered features and CNN features improved dramatically as shown in Figure 5(b). Both the benign and malignant samples were clearly separable, yet several samples were still misclassified in Figure 5(c).

**Figure 6:**
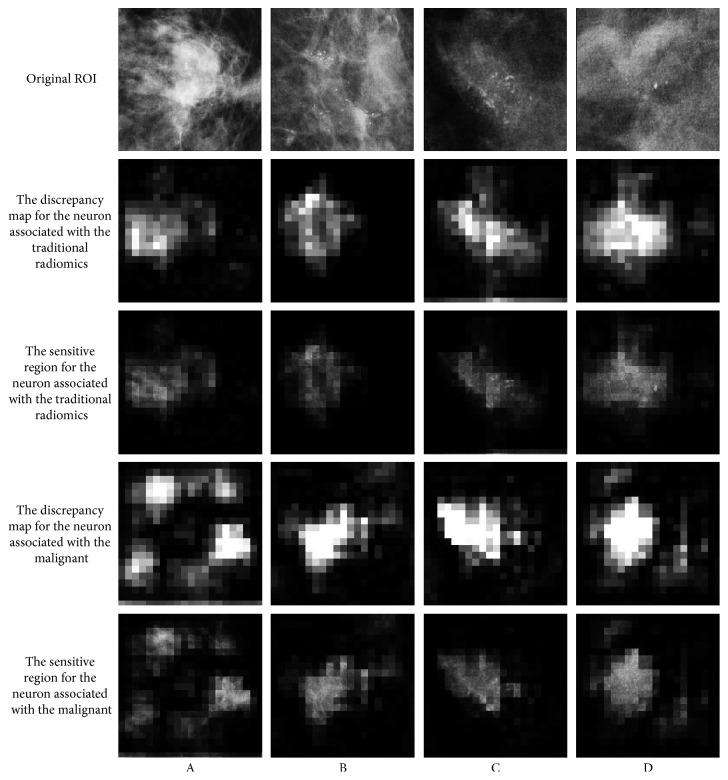
Visualization for the specified neurons.

To further investigate the clinical value of deep features, the detected highly sensitive region found by the neurons were further visualized [42]. We get the neurons with a strong association with traditional radiomics and the neurons with the largest weight of malignant output from the penultimate layer of the whole network. It replicates each ROI many times with small occludes at different locations in the ROI. Then, we feed all of them to the trained network and record the change for the neuron mentioned above. The discrepancy is positively related to the significance of the given patch. As shown in Figure 6, the neurons with a strong association with traditional radiomics (the second and third rows) basically catch the region of MCs. However, it seems that the neurons with the largest weight of malignant output (the fourth and fifth rows) catch the spicule and the lobulation. It is obvious that these features play an important role in diagnosis, but they do not belong to the features of calcification.

## 6. Conclusions

In this study, we have made advances toward the end-to-end training of a deep CNN for microcalcification discrimination for breast cancer screening. The images were collected from two distinct medical institutions. We demonstrated that it is essential to perform feature selection for deep features with the help of manual features. To compare traditional methods using the same standard, we employed our trained model to extract deep features and verified the capacity for deep CNN to capture similar characteristics to that of manual features. Our results showed that CNN is powerful enough to discriminate microcalcification. Furthermore, our proposed method verified that traditional morphological features could be useful to guide CNN features to achieve higher accuracy for classification of MCs.

In the current study, the ROI extraction was done by using an automatic processing method. In [43], the authors introduced a mass detection model based on RetinaNet [44], which is a state-of-the-art one-stage object detector. The experimental evaluation suggests that the model could be used in different patient groups. Indeed, our experimental results showed that the traditional ROI region extraction method had its weaknesses. Therefore, we will consider the ROI region extraction and lesion classification to be automatically implemented by the CNN method in the future work.

## Figures and Tables

**Table 1:** Number of mammograms with malignant and benign pathologies.

Pathology types	Sample size
*Malignant*	**540**
Ductal carcinoma in situ	194
Invasive ductal carcinoma (IDC)	145
Invasive lobular carcinoma (ILC)	5
Breast adenopathy	162
Ductal carcinoma in situ with microinvasion	22
Ductal carcinoma in situ with localized infiltration	5
Mixed types	16
Invasive carcinoma	11
*Benign*	**450**
Fibrocystic mastopathy	297
Inflammation	58
Fibroadenoma	58
Benign lesion for follow-up	14
Benign phyllodes tumor	23

**Table 2:** Overview of the number of images.

	Training images	Augmented training images	Test images
Benign	405	1620	45
Malignant	486	1944	54

**Table 3:** Comparison of performance for MC classification on different sets of features.

Method	Accuracy	Precision	Specificity	AUC	Sensitivity
CNN	0.8768 ± 0.0431	0.8891 ± 0.0349	0.8667 ± 0.0457	0.9336 ± 0.0238	0.8701 ± 0.0144

Morphological	0.8525 ± 0.0203	0.8624 ± 0.0267	0.8311 ± 0.0471	0.9256 ± 0.0211	0.8492 ± 0.0246
CNN + morphological	0.8828 ± 0.0437	0.8911 ± 0.0447	0.8667 ± 0.0602	0.9385 ± 0.0238	0.8761 ± 0.0104
CNN filtered by morphologic	**0.8859** **±** **0.0363**	**0.8932** **±** **0.0384**	**0.8689** **±** **0.0528**	0.9392 ± 0.0240	**0.8843** **±** **0.0344**

Textural	0.7677 ± 0.0634	0.7964 ± 0.0659	0.7511 ± 0.0924	0.8721 ± 0.0530	0.7703 ± 0.0544
CNN + textural	0.8727 ± 0.0500	0.8853 ± 0.0410	0.8622 ± 0.0522	0.9338 ± 0.0248	0.8801 ± 0.0434
CNN filtered by textural	0.8747 ± 0.0387	0.8842 ± 0.0423	0.8578 ± 0.0603	**0.9434** **±** **0.0220**	0.8831 ± 0.0276

Morphological + textural	0.8667 ± 0.0223	0.8768 ± 0.0309	0.8489 ± 0.0511	0.9381 ± 0.0219	0.8601 ± 0.0251
CNN + morphological + textural	0.8818 ± 0.0434	0.8895 ± 0.0457	0.8644 ± 0.0624	0.9379 ± 0.0237	0.8791 ± 0.0124
CNN filtered by morphological + textural	0.8747 ± 0.0376	0.8873 ± 0.0238	0.8644 ± 0.0339	0.9398 ± 0.0242	0.8751 ± 0.0328

**Table 4:** Performance comparisons under various cross-validation ratio.

	90% vs 10%	80% vs 20%	75% vs 25%
Accuracy	0.818	0.791	0.777
Precision	0.865	0.834	0.829
AUC	0.820	0.792	0.780

**Table 5:** Comparison of performances on other models reported early.

Method	Dataset	Accuracy (%)
L-ISOMAP + SSAE [37]	BreaKHis	99.4
Deep features + CNN [38]	BreaKHis	83.3
CSDCNN [39]	BreaKHis	94.9
Grassmannian + VLAD [40]	BreaKHis	90.5

## Data Availability

The data used to support the findings of this study are available from the corresponding author upon request.

## Supplementary Materials

Supplementary Materials The spatial and morphological measurements of MCs include one- and two-dimensional statistics that consist of 156 features. We not only calculated such features for the segmented MCs but also dilated in the different scale as the MC region to describe the distribution and the density shown in Figure S1. For each dilated region, we can extract 31 features. The specific feature illustration is listed in Supplemental Table S1.
