# Supplementary material for: Breast Microcalcification Diagnosis Using Deep Convolutional Neural Network from Digital Mammograms
Source: Comput Math Methods Med. 2019 Mar 3;2019:2717454. doi: 10.1155/2019/2717454 (PMC6421727; doi:10.1155/2019/2717454)
Supplement: Supplementary Materials — The spatial and morphological measurements of MCs include one- and two-dimensional statistics that consist of 156 features. We not only calculated such features for the segmented MCs but also dilated in the different scale as the MC region to describe the distribution and the density shown in Figure S1. For each dilated region, we can extract 31 features. The specific feature illustration is listed in Supplemental Table S1. [file 2717454.f1.pdf]

Supplementary Materials

Spatial and morphological features

The spatial and morphological measurements of MCs include one and two-dimensional statistics that consist of 156 features. We not only calculated such features for the segmented MCs, but also the dilated in the different scale as MC region to describe the distribution and the density as Figures S1 shown. For each dilated region, we can extract 31 features. The specific feature illustration is listed in Supplemental Table S1.

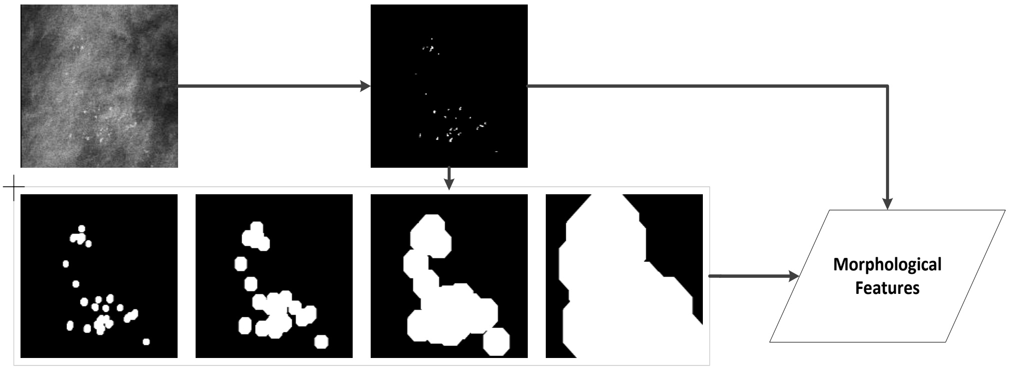

Figures S1: Shown is the quantization of spatial and morphological features. Dilation operation is performed in the segmented calcification. Then the morphological features are extracted from both the segmented and the dilated region.

Texture features

The texture is estimated by two popular methods, including GLCM, and GLRLM. The GLCM is calculated by counting the number of times adjacent pixels have the same orientation. We construct sixteen GLCM matrix in different distance {1, 3, 5, 7} and different direction {0, 45, 90, 135}. At last, 352 GLCM features were extracted for each segmented ROI, including autocorrelation, contrast, correlation, cluster prominence, cluster shade, dissimilarity, energy, entropy, homogeneity, maximum probability, sum of squares, sum average, sum variance, sum entropy, difference variance, difference entropy, information measure of correlation 1, information measure of correlation 2, inverse difference, inverse difference normalized, and inverse difference moment normalized for each GLCM matrix.

The GLRLM is calculated by the number of adjacent pixels showing the same gray intensity from the reference pixels in a particular direction. We construct four GLRLM matrix in different direction {0, 45, 90, 135}. 44 GLRLM features were estimated, including Short-Run Emphasis, Long-Run Emphasis, Gray-Level Non-Uniformity, Run Length Non-Uniformity, Run Percentage, Low Gray-Level Run Emphasis, High Gray-Level Run Emphasis, Short-Run Low Gray-Level Emphasis, Short-Run High Gray-Level Emphasis, Long-Run Low Gray-Level Emphasis, and long-run high Gray-Level Emphasis for each GLRLM matrix.

Table S1: Morphological feature illustration.

<table>
<tr><th>Definition</th><th>Quantitative Way</th><th>Definition</th><th>Quantitative Way</th></tr>
<tr><td>Number of MCs/MC region</td><td>-</td><td>Area of MC/MC regions</td><td>Calculate the sum of the area of MCs/MC regions</td></tr>
<tr><td>Mean major axis for the MCs/MC regions</td><td>Calculate the mean for the major axis length of the MCs/MC regions</td><td>Mean minor axis for the MCs/MC regions</td><td>Calculate the mean for the minor axis length of the MCs/MC regions</td></tr>
<tr><td rowspan="3">The shape of the MCs/MC regions</td><td>Calculate the number/proportion of the MCs/MC regions whose eccentricity less than 0.3</td><td rowspan="3">Shape of the MCs/MC regions</td><td>Calculate the number/proportion of the MCs/MC regions whose extent less than 0.3</td></tr>
<tr><td>Calculate the number/proportion of the MCs/MC regions whose eccentricity more than 0.3 and less than 0.7</td><td>Calculate the number/proportion of the MCs/MC regions whose extent more than 0.3 and less than 0.7</td></tr>
<tr><td>Calculate the number/proportion of the MCs/MC regions whose eccentricity more than 0.7</td><td>Calculate the number/proportion of the MCs/MC regions whose extent more than 0.7</td></tr>
<tr><td rowspan="4">The shape of the MCs/MC regions</td><td>Calculate the number/proportion of the MCs/MC regions whose solidity less than 0.3</td><td rowspan="4">Direction of the MCs/MC regions</td><td>Calculate the number/proportion of the MCs/MC regions whose orientation less than -45</td></tr>
<tr><td rowspan="2">Calculate the number/proportion of the MCs/MC regions whose solidity more than 0.3 and less than 0.7</td><td>Calculate the number/proportion of the MCs/MC regions whose orientation more than -45 and less than 0</td></tr>
<tr><td>Calculate the number/proportion of the MCs/MC regions whose orientation more than 0 and less than 45</td></tr>
<tr><td>Calculate the number/proportion of the MCs/MC regions whose solidity more than 0.7</td><td>Calculate the number/proportion of the MCs/MC regions whose orientation more than 45</td></tr>
<tr><td>Mean diameter for the MCs/MC regions</td><td>Calculate the mean diameter for the MCs/MC regions</td><td>The scope of the calcification cluster</td><td>Calculate the area of the ROI</td></tr>
</table>
